# Prof. Moore’s Clinic at Buffalo General Hospital

**Published:** 1881-04

**Authors:** 


### PROF. MOORE'S CLINIC AT BUFFALO GENERAL HOSPITAL.

REPORTED BY DR. PETERSON.

FEB. 16, 1881.

CASE I.—The boy, on whom a plastic operation was made in the early part of the year, to replace his left cheek, which from some ulcerative disease had sloughed out, extending from the mouth to the ramus of the jaw. The flap was taken from the neck and twisted to fill the place. The result is perfect. There is scarcely any deformity.

Case II.—The case of epilepsy examined in Prof. Rochester's clinic this morning. The case is pre-eminently fitted for trephining, but the man being a stranger, found in the streets of Buffalo, it would not be proper to operate upon him without his own consent or that of his friends.

Case III.—(Case V, Feb. number Journal), the man with frozen hands. The fingers are very raw and swollen. Some of the phalanges are exposed and dead, and the periosteum entirely destroyed. Two middle phalanges of the left hand we will remove immediately with forceps. When they are attached at one end, although necrosed at the other, it is best to leave them alone, as nature may throw off a scale of bone and preserve the rest. One of the phalanges of the right hand it is also best to remove. The spaces left vacant by these bones will fill with granulations, and the fingers will be drawn down into a knot. Sometimes a new bone will form.

Case IV.—(Case II, Feb. number Journal, R. G. T.) The drainage tube has been left in, and will be allowed to remain still longer. The fistula is very sensitive. There is no caries above the ramus of the ischium, but a little below, which is very loose, and nature will separate it in a short time. The bladder is somewhat irritated. The patient will recover.

Case V.—Henry Green; aged 54; varicose ulcer; now healed; has been under treatment for some weeks. The ulcer was several inches square, and has been treated by strapping with adhesive plaster, which is the best method. The resin of the plaster is a disadvantage, but the main thing is the pressure. The high edges of the ulcer are pressed down to their proper level, so the skin can extend itself, and the dilated capillary vessels are contracted to their normal calibre. This is the only plan for treating all ulcers on the leg, except syphilitic. Sometimes the sudden healing of an ulcer, which has for many years acted as an issue on a patient, is productive of evil by lowering vital power, as in this case; this is best counteracted by the daily administration of laxatives.

CASE VI.—Male; had ununited fracture of right leg at junction of middle and lower thirds. It was broken Nov. 17, 1880 At the end of six weeks there was no union. Bandages were applied more tightly, and it was recommended to wait. It is now three months and the leg has just united. This is a case of delayed union, and not non-union. It is always best to wait in these cases.

CASE VII.—Jno. Berry; aged 23; leaped from a window at the Birge factory fire, late in December. His back was broken in the middle dorsal region. There was a projection and crepitus. By using a great deal of force he was pulled out straight, laid upon his back, and adhesive plaster and extension applied. He is now perfectly recovered. This is one case in 10,000. It is a wonderful result. The walk is natural, and the spinal cord is healthy. The right humerus was also broken, and an excellent result obtained by means of extension. A fracture of his left forearm was so successfully treated as to secure to him perfect pronation and supination.

CASE VIII.—Male; aged 30; had rheumatism six years; one year ago some one prescribed colchicum, black cohosh and guaiac in large doses, and he was suddenly taken with convulsions, which resulted in anchylosis of both knees, with the feet bent inwards. Patient attributes deformity to medicine. The professor finds that a clergyman had administered it. Clergymen should not meddle with medicine. They are sharp enough in other things, but not in this. Where there are convulsions, should always examine urine for albumen, and if you find it, give Epsom salts, the most trustworthy remedy. In this case the muscular condition is curious. The toes are turned in by the adductors of the legs, as pronators often act in forearm upon the thumbs. Thinks the anchylosis ought to be destroyed, even at the risk of breaking the legs. Will not undertake it to-day.

CASE IX.—Male; aged 20; left hand perforated in the centre by an arrow. Has been shown the class before. A drainage tube has been kept in it sometime, but may now be removed. On probing find there is no dead bone. Will heal soon.

CASE X.—Girl; aged 8; varus equinus of both feet; was operated upon before the class some time ago. The result is gratifying. The feet can easily be brought to their proper angle.

CASE XI.—(Case II, Jan. 19, Feb. number JOURNAL). The little child is doing well. The plaster of Paris bandages are now kept on the right leg. His general health is good.

CASE XII.—Female; aged 30; necrosis of vomer and nasal cartilage. Would suspect syphilis, but patient denies it, and the professor believes her. It is lupus exedens. Lupus is of a bright red color while the syphilitic ulcer is of a dark red. There is no sore throat nor sore mouth. It began on the nose. Lupus generally selects the nose, and it is more usual to see it earlier in life, but adults are sometimes affected. This began at the tip of the nose and extended. The therapeutics of lupus are unsatisfactory. The use of either pot. jodidum or hydrargyrum is a failure. The best and only remedy is arsenic. The general health should be carefully attended to. Advises Fowler's Sol. gii. v. t. i. d. Local treatment is of no avail. This case is one of the most interesting of the winter.

CASE XIII.—John D.; aged 19; condition at present: contraction of fingers of right hand into palm from injury to nerves and tendons from four fractures in that forearm; ankylosis of left knee, necrosis in left thigh bone from the compound comminuted fracture of that bone in 1879. For a report of this remarkable case see BUFFALO MEDICAL JOURNAL of March, 1880, "A Fracture Case." There were over seven fractures.

CASE XIV.—Male; aged 30; ankylosis fingers right hand; have been treated so successfully as to gain some flexion.

CASE XV.—Female; aged 6; ulcer of cornea two years ago; there is now a white spot on the cornea of left eye, and also an exudate from an old iritis. It is now leucoma. Ophthalmologists use large names. The smaller the object in natural history, the larger the name. Atropine should have been used there to get the iris out of the way. Now nothing can be done.

CASE XVI.—S.; male; aged 35; unhealed stump. On amputation was performed eighteen months before at metatarsus joint—Hey's operation—and the non-healing is due to the strain on the cicatrix. The remedy for this is proper bandaging for the purpose of relieving the strain, and this treatment has been employed upon this stump for some time with excellent effect.

The Professor then closed the last surgical clinic of the season with thanks to those physicians in the city who had been kind enough to send him cases for the instruction of the students.

